# Carriage of critically important antimicrobial resistant bacteria and zoonotic parasites amongst camp dogs in remote Western Australian indigenous communities

**DOI:** 10.1038/s41598-018-26920-5

**Published:** 2018-06-07

**Authors:** Bertha Rusdi, Tanya Laird, Rebecca Abraham, Amanda Ash, Ian D. Robertson, Shewli Mukerji, Geoffrey W. Coombs, Sam Abraham, Mark A. O’Dea

**Affiliations:** 1 Antimicrobial Resistance and Infectious Diseases Laboratory, School of Veterinary and Life Sciences, Murdoch University, Perth, Australia; 2 Australian Centre for Antimicrobial Resistance Ecology, University of Adelaide, Adelaide, Australia; 3 College of Veterinary Medicine, School of Veterinary and Life Sciences, Murdoch University, Perth, Australia; 4 PathWest laboratory Medicine – WA, Fiona Stanley Hospital, Murdoch, Australia

## Abstract

Camp dogs in indigenous communities in the Western Australian Kimberley Region, share the domestic environment with humans and have the potential to act as carriers of, and sentinels for, a wide range of zoonotic agents, including intestinal parasites and antimicrobial resistant bacteria. In this study, we investigated the carriage of extended-spectrum-cephalosporin-resistant (ESC-resistant) *Escherichia coli*, methicillin-resistant *Staphylococcus aureus* (MRSA) and species of hookworm and *Giardia* among camp dogs in remote Western Australian Aboriginal communities. A total of 141 canine faecal samples and 156 nasal swabs were collected from dogs in four communities of the Western Australian Kimberley region. Overall, ESC-resistant *E. coli* was detected in 16.7% of faecal samples and MRSA was isolated from 2.6% of nasal swabs. Of most significance was the presence of the community-associated Panton-Valentine leucocidin (PVL)-positive MRSA ST93 and ST5 clones and ESC-resistant *E. coli* ST38 and ST131. The most prevalent zoonotic intestinal parasite infection was *Ancylostoma caninum* (66%). The prevalence of *Giardia* was 12.1%, with the main genotypes of *Giardia* detected being dog specific assemblages C and D, which are unlikely to cause disease in humans.

## Introduction

Greater than 60% of emerging human infectious diseases are zoonotic^1^. Companion animals, such as dogs, have been shown to be sources of zoonoses^2^, in part due to close, prolonged contact with humans and integration into environments of increased human population density. Parasitic and bacterial zoonoses are commonly found in household pets^3^. Recently, the prevalence of multidrug resistant *Escherichia coli* resistant to extended spectrum cephalosporins (ESCs), methicillin-resistant *Staphylococcus aureus* (MRSA) and methicillin-resistant *Staphylococcus pseudintermedius* (MRSP) in domestic pets has been increasing^4–9^.

Gram-negative bacteria resistant to critically important antimicrobials (CIAs) such as carbapenems and ESCs are of significant public health concern due to limited therapeutic options and the ability of such antimicrobial resistance to be transferred to sensitive Gram-negative bacteria via horizontal gene transfer^10,11^. It is possible that companion animals may serve as reservoirs for ESC-resistant *E. coli* due to the close associations between humans and pets^12,13^. Studies have reported human and companion animal isolates sharing the same genes and displaying identical clonal lineages^14–17^, suggesting transmission of the bacteria between household pets and humans.

MRSA is a zoonotic and zooanthroponotic agent identified among companion animals and a global health issue. The first human community-acquired MRSA (CA-MRSA) infections were reported in Australian Aboriginal and native Canadian communities in the 1990s^18,19^, and studies have also reported evidence of MRSA transmission between human and companion animals^20–22^.

In addition to the growing threat from CIA resistant bacteria, the public health threat from zoonotic parasites requires close monitoring, particularly among isolated communities of low socioeconomic standing and with poor health education. *Ancylostoma caninum* is a zoonotic canine hookworm species and is one of the soil-transmitted helminths^23^. The most common route of hookworm infection is via penetration of the skin by hookworm larva^24^. As such, communities where the parasite is endemic, coupled with behavior of walking barefoot, increases the risk of infection. Zoonotic infection with *Ancylostoma caninum* can result in cutaneous larva migrans, a creeping, itchy rash caused by migrating larvae under the skin^25^.

*Giardia* is a cause of gastrointestinal infection which is common in humans around the world, particularly in developing regions^26^, and a high prevalence of *Giardia* infection among indigenous communities in Western Australia has been documented^27,28^. Clinical manifestations include diarrhea, nausea, and abdominal pain and distension^25^, and serious sequelae in infants and children include failure-to-thrive syndrome^29^.

Previous studies into zoonotic intestinal parasites from dogs in remote Aboriginal communities in the Kimberley region of Western Australia were conducted over 20 years ago^27,30^, and data on antimicrobial resistant (AMR) bacteria in companion animals in these communities is lacking. This study provides a contemporary examination of the zoonotic parasites and antimicrobial resistant bacteria from camp-dogs in Aboriginal communities in the Kimberley region.

## Results

### Carriage of ESC-resistant *E. coli*

ESC-resistant *E. coli* was isolated from 12.8% (18/141) of faecal samples, with the majority coming from the West Kimberly communities (12 of 21 isolates). The 18 positive faecal samples yielded, in total, 21 ESC-resistant *E. coli* isolates.

The Isolates were defined by multi-locus sequence-type (MLST) with most isolates belonging to sequence type (ST) 38 (42.8%, n = 9). The remaining isolates were characterised as ST2144 (9.5%, n = 2), ST131 (9.5%, n = 2), ST1408 (4.3%, n = 1), ST3520 (4.3%, n = 1), ST1569 (4.3%, n = 1), ST68 (4.3%, n = 1), ST3268 (4.8%, n = 1), ST106 (4.8%, n = 1) and ST872 (4.8%, n = 1). The two ST131 isolates were identified in the East Kimberley 2 (EK2) region while the ST2144 isolates were from West Kimberley 1 (WK1) region. As a result of low sequence coverage, the STs for two isolates could not be determined.

All ESC-resistant *E. coli* isolates were resistant to two or more classes of antimicrobials (multidrug resistant) with universal resistance to ceftriaxone and ampicillin. Two of the isolates from EK2, (ST38 and ST131), and one ST3268 isolate from East Kimberley 3 (EK3) demonstrated resistance or intermediate resistance to ciprofloxacin. The ST38 and ST131 isolates were also resistant to trimethoprim/sulfamethoxazole. Two ST38 isolates, from different locations, were resistant to six or more antimicrobial classes and carried corresponding resistance genes. The two phenotypically and genotypically distinguishable ST2144 were isolated from the same animal with additional resistance to cefoxitin and amoxicillin-clavulanate (Table 1).

**Table 1:** Genotypic and phenotypic antimicrobial resistance profile of ESC-resistant *E. coli* isolates collected from dog faeces in remote Australian communities.

Isolate	Origin	MLST	Resistance Genes	Phenotypic resistance
B1 HC6	EK 2	ST131	*aadA5, mph(A), sul1, dfrA17 bla*_CTX-M-15_, *gyrA* (S83L, D87N, E678D), *gyrB* (D185E), *parC* (S80I, E475D)	CRO, SXT, AMP, CIP
B1 HC22	EK 2	ST131	*aadA5, mph(A), sul1, dfrA17 bla*_CTX-M-27_*, bla*_TEM-1B_*, aac(3)-IId strB, strA, sul2, tet(A)*, *gyrA* (S83L, E678D), *gyrB* (D185E)	TET, CRO, CN, SXT, AMP, S
B1 HC19	EK 2	ST38	*aadA5, mph(A), sul1, dfrA17, bla*_CTX-M-14_, *bla*_TEM-1B_*, aac(3)-IId, strB, strA, sul2, tet(D), catA1, gyrA* (S83L, S828A), *gyrB* (D185E), *parC* (S80I), *parE* (I136V)	TET, CRO, CN, SXT, C, AMP, S
B1 HC10	EK 2	ST1408	*mph(A), bla*_CTX-M-14_*, tet(A*)	TET, CRO, AMP
B2 B2	WK 1	ST3520	*aadA5, mph(A), sul1, dfrA17, bla*_CTX-M-27_	CRO, SXT, AMP
B2 B4.A	WK 1	ST2144	*bla*_CMY-2_	CRO, AMP, FOX, AMC
B2 B4.B	WK 1	ST2144	*bla*_TEM-33_ *, bla*_CMY-2_	CRO, AMP, FOX,, AMC
B2 B10.A	WK 1	ST38	*aadA5, mph(A), sul1, dfrA17, bla*_CTX-M-14_, *bla*_TEM-1B_*, aac(3)-IId, strB, strA, sul2, tet(D), catA1, gyrA* (S83L, S828A), *gyrB* (D185E), *parC* (S80I), *parE* (I136V)	TET, CRO, CN, SXT, C, AMP, S, AMC*
B2 B10.B	WK 1	ST38	*aadA5, mph(A), sul1, dfrA17, bla*_CTX-M-27_*, gyrA* (S828A), *gyrB* (D185E), *parE* (I136V)	CRO, SXT, AMP
B2 B12.A	WK 1	ST38	*aadA5, mph(A), sul1, dfrA17, bla*_CTX-M-27_, *gyrA* (S828A), *gyrB* (D185E), *parE* (I136V)	CRO, SXT, AMP
B2 B13	WK 1	ST38	*aadA5, mph(A), sul1, dfrA17, bla*_CTX-M-27_, *gyrA* (S828A), *gyrB* (D185E), *parE* (I136V)	CRO, SXT, AMP
B2 B14	WK 1	ST38	*aadA5, mph(A), sul1, dfrA17, bla*_CTX-M-27_, *gyrA* (S828A), *gyrB* (D185E), *parE* (I136V)	CRO, SXT, AMP
B2 B17.A	WK 1	ST38	*aadA5, mph(A), sul1, dfrA17, bla*_CTX-M-27_, *gyrA* (S828A), *parE* (I136V)	CRO, SXT, AMP
B2 B17.B	WK 1	ST1569	*dfrA17, bla*_CTX-M-27_, *gyrA* (S828A), *gyrB* (D185E), *parE* (I136V)	CRO, SXT, AMP
B2 B18	WK 1	ST-38	*aadA5, mph(A), sul1, dfrA17, bla*_CTX-M-27_	CRO, SXT, AMP
B2 B22	WK1	ST68	*QnrS1, bla*_CTX-M-15_	CRO, AMP
K2–10	EK3	ST3268	*QnrS1, dfrA14, bla*_TEM-1B_*, bla*_CTX-M-15_, *gyrA* (S828A)	TET, CRO, AMP, CIP*
K1–22	EK2	ST38	*aadA5, sul2, dfrA17, bla*_CTX-M-14_*, bla*_TEM-1B_*, aac(3)-IId, strB, strA, tet(D), catA1*, *gyrA* (S83L), *gyrB* (D185E), *parC* (S80I), *parE* (I136V)	AMP, SXT, TET, C, AMC*, S, CN, CIP*, CRO
K1–38	EK2	Unknown	*sul1, dfrA17, bla*_CTX-M-27_	AMP, SXT, CRO
K1–39	EK2	ST106	*aadA5, mph(A), dfrA17, bla*_CTX-M-14_ *, cmlA1, erm(B)*	AMP, SXT, S, CRO
K1–41	EK2	Unknown	*sul2, QnrS1, bla*_CTX-M-11_ *, bla*_TEM-1B_	AMP, CRO

EK (East Kimberley), WK (West Kimberley), *(Intermediate), SXT (Trimethoprim/Sulfamethoxazole), TET (Tetracycline), FOX (Cefoxitin), CRO (Ceftriaxone), CN (Gentamicin), C (Chloramphenicol), AMP (Ampicillin), S (Streptomycin), CIP (Ciprofloxacin), AMC (Amoxicillin-clavulanate).

All sequenced isolates were found to carry beta lactam resistance genes: *bla*_CTX-M27_ (n = 10), *bla*_CTX-M14_ (n = 5), *bla*_CMY-2_ (n = 3), *bla*_TEM-1B_ (n = 6), *bla*_CTX-M15_ (n = 3), and *bla*_TEM-33_ (n = 1). Several other antibiotic class resistance genes were also carried by the isolates; including trimethoprim (*drfA17*), sulphamethoxazole (*sul1*/*sul2*), macrolide (*mph)*, erythromycin (*erm*), quinolone (*qnrS1*), chloramphenicol (*cat A1*) and tetracycline (*tetA*/*tetD*). Thirteen isolates harbored the macrolide resistance gene *mph(A)*. Aminoglycoside resistance genes detected were *aadA5* (n = 13), *strA*/*strB* (n = 4), and *aac(3)-IId* (n = 4). A tetracycline resistance gene (*tetA* or *tetD*) was detected in five isolates. Quinolone resistance (*qnrS1*) and chloramphenicol resistance (*catA1*) genes were also found in three isolates each. The erythromycin resistance gene (*ermB*) was harbored by one isolate.

### Carriage of MRSA

MRSA was isolated from 2.6% (4/156) of the nasal swabs, two isolates from EK1, one isolate from EK2 and one isolate from WK1. The four MRSA isolates were cefoxitin and penicillin resistant, but trimethoprim/sulfamethoxazole, tetracycline, erythromycin, ciprofloxacin and gentamicin susceptible (Table 2). Two of the four MRSA isolates were identified as ST93. The remaining two isolates were identified as ST5 and ST872 (Table 2). All isolates carried beta-lactam (*blaZ)*, methicillin (*mecA)* and efflux pump (*norA*) genes. The Panton Valentine leucocidin (PVL) associated genes, *lukF-PV and lukS-PV*, were detected in 3 of 4 isolates (ST93 and ST5).

**Table 2:** Molecular characteristics and phenotypic antimicrobial resistance profiles of nasal MRSA isolates collected from dogs in remote Australian communities.

Isolate	Origin	Sequence type	Resistance genes	Phenotypic antimicrobial resistance	Virulence Factors
B1 B19	EK 1	ST5	*blaZ, mecA, norA*	FOX, P	*aur, splB, splA, scn, sak, lukF-PV, lukS-PV, hlb, hlgB, hlgA, hlgC, lukD, lukE, edinA, seg, sen, seu, sei, sem, seo*
B1 B21	EK 1	ST93	*blaZ, mecA, norA*	FOX, P	*splA, aur, splE, scn, sak, lukS-PV, lukE, hlb, hlgA, hlgC, hlgB, lukF-PV, lukD*
B2 B61	WK 1	ST93	*blaZ, mecA, norA*	FOX, P	*splA, splE, aur, scn, sak, hlb, lukF-PV, lukS-PV, lukE, lukD, hlgA, hlgC, hlgB*
B1 HC17	EK 2	ST872	*blaZ, mecA, norA*	FOX, P	*splB, splA, aur, splE, sak, scn, lukD, lukE, sea/sep, hlgA, hlgC, hlgB, seq, sek, hlb, seh*

EK 1 (East Kimberley, Community 1), EK 2 (East Kimberley, Community 2), WK 1 (West Kimberley, Community 1), FOX (cefoxitin), P (penicillin).

### Carriage of zoonotic parasites

Based on molecular detection results of the species of interest, 82 dogs were infected with hookworm (*A. caninum*) alone, six dogs were infected with *Giardia* alone, and 11 dogs were infected with hookworm and *Giardia* combined. A total of 111 dogs were found to be infected with one or more parasites based on combined molecular and microscopic examination. Overall, hookworm infection was the most common with 93 of 141 dogs (66%) positive. The other helminths observed in the samples were *Toxocara canis* (4.3%, n = 6), *Spirometra erinaceid* (3.5%, n = 5), *Taenia spp*. (1.4%, n = 2), and *Spirocerca lupi* (0.7%, n = 1). *Giardia* was the second most prevalent protozoa in dogs (12.1%, n = 17) after *Isospora spp*. (12.7%, n = 18) followed by *Sarcocystis spp*. (9.9%, n = 14). The prevalence of *Giardia* and hookworm in each community in the Kimberley region is shown in Table 3. EK1 had the highest crude prevalence of hookworm (93.8%) and *Giardia* (18.8%).

**Table 3:** Prevalence of *Giardia* and hookworm in canine faeces collected in the Kimberley region.

Origin	*Ancylostoma caninum*	Prevalence (95% CI)	*Giardia spp*.	Prevalence (95% CI)
EK 1	15/16	93.8% (71.7, 98.9)	3/16	18.8% (6.6, 43.0)
EK 2	46/71	64.8% (53.2, 74.9)	9/71	8.5% (6.8, 22.4)
EK 3	12/26	46.2% (28.8, 64.5)	2/26	7.7% (2.1, 24.1)
WK 1	20/26	76.9% (57.9, 90.0)	3/26	11.5% (4.0, 29.0)
WK 2	0/2	0% (0.00, 65.8)	0/2	0% (0.00, 65.8)
Total	93/141	66%(57.8,73.3)	17/141	12.1% (7.7, 18.5)

Most dogs from which sequencing was successful (12/17 dogs, 70.6%%) were carrying *Giardia* Assemblage C (GenBank accession numbers: MF974555-MF974558; MF974560, MF990014- MF990016, MF769400). Three Isolates; B1 HC10 (GenBank accession number: MF990017), K2–27 (100% similar to GenBank accession number: KY979492) and B1 HC34 (GenBank accession number: MF990018) were identified as assemblage D at the *gdh* locus. Evidence suggesting mixed populations was found in two isolates, B1 HC23 and B1 HC7. Isolate B1 HC23 was identified as assemblage D at the *gdh* locus and assemblage C (GenBank accession number: MF974560) at the *tpi* locus. The sequence of Isolate B1 HC7 at the *gdh* locus matched representative GenBank accessions for assemblage C using the forward primer for Sanger sequencing and assemblage D when using the reverse primer. At the *tpi* locus the sequence was identified as assemblage A (GenBank accession number: MF974560) (Table 4).

**Table 4:** *Giardia* assemblages identified in dog faeces based on combined sequencing data from *gdh* and *tpi* genes. NS: No sequence determined.

Isolate	*Gdh*	*tpi*	Combined result
	Assemblage	Assemblage	Assemblage
B1 B12	C	C	C
B2 B12	C	C	C
B2 B18	C	NS	C
B2 B25	NS	C	C
B1 B5	C	C	C
B1 B7	C	C	C
B1 HC10	D	NS	D
B1 HC23	D	C	D + C
B1 HC34	D	NS	D
B1 HC7	C + D	A	C + D + A
K1–2	C	NS	C
K1–5	C	NS	C
K1–31	C	C	C
K1–34	C	C	C
K1–41	C	C	C
K2–13	C	C	C
K2–27	D	NS	D

## Discussion

The current study provides information on the carriage of ESC-resistant *E. coli*, MRSA and zoonotic enteric parasites among camp dogs that are in close contact with Western Australian Aboriginal communities. ESC-resistant *E. coli* were carried by 16.7% of dogs with some of the isolates belonging to the globally disseminated ST131 and ST38 ESC-resistant pandemic clones. Additionally, camp dogs were colonized by PVL-positive CA-MRSA (ST93 and ST5) clones at a low prevalence (2.6%); and zoonotic intestinal parasites *Giardia* and *Ancylostoma caninum* were present at prevalences of 12.1% and 66% respectively. It should be noted that the prevalences reported are crude, given the opportunistic nature of sampling, and that the numbers of dogs present in each region are only estimates, with no valid enumeration data available.

ST38 was identified as the major *E. coli* ST in the faecal samples collected in two community locations. ST2144 and ST131 were also identified for more than one isolate. The two ST2144 were isolated from the same animal in the West Kimberly community. This sample grew 3 morphologically distinguishable colony types, however whole genome sequencing showed that only two separate genotypes were present. ST131 carrying *bla*_CTX-M_ genes, which was isolated from two animals located in the East Kimberley community, can exist as a globally disseminated multi-drug resistant pandemic extra intestinal pathogenic *E. coli* responsible for causing variety of extra intestinal infections in humans, including urinary tract infection and bacteraemia^31,32^. Significantly, ST131 and ST38 have previously been reported as causes of disease in various animals, including dogs^33–36^. The limitations of this study do not allow conclusions to be drawn on how the sampled animals acquired these infections. It could be hypothesized that they naturally circulate in dogs in these communities or alternatively they are spillover from the human population. However, these findings are of public health concern, given the possibility that these clonal types may be transferred from dogs to humans, and a larger scale study inclusive of human sampling may aid in determining the ecology of resistant *E. coli* in these populations.

PVL-positive ST93 -IV and PVL-negative ST5 -IV are community acquired (CA)-MRSA that have also been found in animals^37,38^. ST93-IV is the dominant CA-MRSA clone across Australia in humans, and has been associated with a range of skin and soft tissue infections, as well as severe invasive infections such as necrotizing pneumonia^38–40^. The three MRSA isolates harbored the beta-lactamase gene (*blaZ)*, the penicillin-binding protein, PBP 2a gene (*mecA*) and the efflux pump gene (*norA*). This finding is of important public health significance in these populations, as these isolates are resistant to beta-lactam antibiotics which may be used for treatment of pneumonia, skin and ear infections that are highly prevalent among Aboriginal communities^41,42^. As for *E coli*, a more detailed study to examine the ecology of these MRSA clones in Aboriginal communities and camp dogs is warranted.

The prevalence of *Ancylostoma caninum* in dogs identified in the current study is similar to a previous report from the same area, completed in 1993^27^. The high prevalence of *A. caninum* increases the opportunity for spread of the infection to humans in the communities, which can cause cutaneous larva migrans^43^. Although *A. caninum* is a zoonotic agent, it is considered of minor public health significance as this species of hookworm rarely progresses past cutaneous infections^23^. Dog Health Programs in Aboriginal communities that were first introduced in the Kimberley region of Western Australia in 1992^44^, used the anthelmintic Ivermectin to reduce the prevalence of scabies and hookworm in dogs. Unfortunately, the treatment was only able to reduce the intensity of the infection but did not significantly diminish the prevalence of canine hookworm^44^. The failure of eradication of the parasite might be correlated to periodic treatment, however, more recently dogs in these communities have received 3-monthly moxidectin treatments. As such these results are of concern, and may indicate anthelmintic resistance or heavy environmental contamination by dogs which have missed regular treatments.

The prevalence of *Giardia* infection in dogs in this study (12.1%, 95% CI 7.7, 18.5) was similar to findings in the same region over 20 years ago (17%)^27^ and to a national study of gastrointestinal parasites of dogs in Australia (9.3%, 95% CI 7.8–10.8)^45^. This study found that the genotype of *Giardia* from dogs in the region were mostly canine-specific Assemblages C and D. The zoonotic *Giardia* Assemblage A was only found in one sample, and it would appear that the likelihood of transmission of *Giardia* between dogs and humans in the Kimberley Region remains low.

The management of dogs is of paramount importance in minimizing the spread of zoonotic agents through these communities. Dogs in this study were able to roam freely, and scavenged on human waste. Access to materials such as human faeces has the potential for dogs to become infected with human associated bacterial clones and parasites, and maintain them in the community. Ongoing de-sexing and treatment clinics together with continuous client education regarding good husbandry practices and correct anthelmintic, antiprotozoal and antibiotic administration are also important to prevent recurrent infections.

In conclusion, this study demonstrates the carriage of antimicrobial resistant bacteria and zoonotic enteric parasites amongst camp dogs in remote Western Australian communities. The carriage of human associated MRSA (ST93 and ST5) and ESC-resistant *E. coli* (ST131 and 38) identified in this study is of particular importance, and requires further study to determine whether there is movement of CIA resistant bacteria from humans to animals and the potential for zoonotic transmission to humans.

## Methods

### Study area

The Kimberley Region is in the north of Western Australia. The region is a remote area populated by Aboriginal communities. The samples were collected from three communities in the East Kimberley and two communities in the West Kimberley. Available information about total dog population and number of samples in each community are presented in Table 5.

**Table 5:** Canine faecal samples by community and population.

Origin	Approximate population size of dogs	Dogs sampled
East Kimberley (EK) 1	30	16
East Kimberley (EK) 2	unknown	71
East Kimberley (EK) 3	unknown	26
West Kimberley (WK) 1	100	26
West Kimberley (WK) 2	60	2

### Source of isolates

Work undertaken in this survey was approved by the Murdoch University Animal Ethics Committee (Permit #408 and #R2876/16), with all experiments performed in accordance with relevant guidelines and regulations. Nasal swabs were collected from 156 dogs from five communities. Of these dogs, faecal samples could be collected from 141, with the remaining having empty rectums. Sampling was conducted for diagnostic purposes by the Murdoch University veterinary team undertaking a neutering operation and dog health program in the Kimberley region in three-time periods; June 2016, October 2016 and June 2017. Sample numbers were based solely on dogs entering the neutering programme. Only dogs which had not been previously neutered were sampled to prevent resampling the same individual. Faecal samples were collected into standard 70 ml plastic containers. Nasal swab samples were collected using swabs into charcoal media (Copan, Italy). Samples were stored at 4 °C until processed.

### Bacterial Isolation and detection

For MRSA isolation, swabs were plated onto Brilliance MRSA Agar (ThermoFisher Scientific) and incubated overnight at 37 °C. Colonies resembling MRSA were subcultured onto 5% Sheep Blood Agar (Edwards Media). Screening for ESC-resistance was performed by incubating the faecal samples onto Brilliance ESBL Agar (ThermoFisher Scientific) and incubating overnight at 37 °C. Colonies resembling ESBL *E. coli* were sub-cultured onto 5% Sheep Blood Agar (Edwards Media). If more than one colony morphology was identified on a plate an isolate from each colony type was taken. Identification of all isolates was conducted using a Bruker microflex MALDI-TOF.

### Antimicrobial susceptibility testing

Isolates underwent susceptibility testing via disc diffusion according to the Clinical Laboratory Standards Institute (CLSI) Performance Standards for antimicrobial disk susceptibility tests M02-A12^46^. MRSA were tested using the following seven antimicrobials: trimethoprim/sulfamethoxazole, tetracycline, cefoxitin, erythromycin, penicillin, ciprofloxacin and gentamicin. *E. coli* isolates were tested using the following 12 antimicrobials: Trimethoprim/Sulfamethoxazole, tetracycline, cefoxitin, ceftriaxone, gentamicin, chloramphenicol, ampicillin, streptomycin, imipenem, ciprofloxacin, amoxicillin-clavulanate and meropenem. Zone diameter results were categorized as susceptible, intermediate and resistant using the clinical interpretative criteria specified in CLSI performance standard VET01-S3^47^. If interpretive criteria was not present in VET01-S3, CLSI performance standard M100-S25 was used^48^.

### Detection of resistance genes

DNA was extracted from isolates using a MagMax DNA multi sample kit (ThermoFisher Scientific) as per manufacturer’s instructions with the modification to omit the RNAse treatment step. Library preparation was performed using an Illumina NexTera XT library preparation kit as per manufacturer’s instructions. Sequencing was performed on an Illumina MiSeq platform using a V3 2 × 300 kit. Reads were de novo assembled using CLC Genomics Workbench v9.5.4, and contig files uploaded to the Centre for Genomic Epidemiology (http://www.genomicepidemiology.org/) for screening for MLST, serotype, antimicrobial resistance genes, plasmid replicon type and virulence factors. SNPs within the quinolone-resistance determining regions of ST38 and ST131 isolates were identified using the Snippy programme within the Nullarbor bioinformatics pipeline^49^.

### Parasite egg identification and DNA extraction

One gram of each faecal sample was examined for the presence of parasite eggs using flotation in saturated zinc sulphate, followed by examination under a light microscope. Briefly, approximately 1 g of faeces was mixed with 9 mL of zinc sulphate solution (specific gravity 1.18) in a 10 mL centrifuge tube. The tube was centrifuged at 900 xg for five minutes with no brake. Additional zinc sulphate solution was added to form a positive meniscus and a cover slip was placed on the top of the tube. After approximately 5 minutes the cover slip was placed onto a slide and examined for the presence of parasites at 100× and 400× magnification. DNA was extracted directly from all faecal samples using a Bioline Isolate II Faecal DNA Kit (Bioline), as per the manufacturer’s instructions. DNA extracts were stored at −20 °C until required.

### Polymerase chain reaction

DNA extracts were subject to qPCR for identification of *Giardia*, conventional PCR for differentiating *Ancylostoma* species and conventional PCR for genotyping *Giardia*. An approximately 380 bp section of internal transcribed spacer-2 (ITS-2) region of *Ancylostoma spp*. was amplified using a protocol modified from Smout *et al*.^50^. Primers used in this assay are listed in Table 6. The PCR reaction mix was prepared in a volume of 25 µL consisting of 12.5 µL GoTaq® Green Master Mix (Promega,USA), 0.25 µM of each primer, 6.25 µL nuclease-free water and 5 µL of template genomic DNA. The thermocycling conditions consisted of a pre-heating step at 94 °C for 2 min, followed by 40 cycles of 94 °C for 30 s, 64 °C for 30 s, 72 °C for 30 s, and a final extension of 72 °C for 7 min. PCR products were viewed on a 1.5% agarose gel dyed with SYBR®Safe DNA gel stain.

**Table 6:** PCR primers for *Ancylostoma spp*. and *Giardia*.

Target	Primer name	Sequence	Reference
***Ancylostoma spp*** *.*	RTHWIF	5′-GATGAGCATTGCWTGAATGCCG-3′	^50^
	RTHWIR	5′-GCAAGTRCCGTTCGACAAACAG-3′	
***Giardia gdh***			
Conventional PCR	Primary reaction		^52,^ ^53^
	*GDH*eF	5′TCAACGTYAAYCGYGGYTTCCGT-3′	
	*GDH* 2	5′-ACCTCGTTCTGRGTGGCGCA-3′	
	Secondary reaction		
	*GDH*iF	5′-CAGTACAACTCYGCTCTCGG-3′	
	*GDH* 4	5′-GTGGCGCARGGCATGATGCA-3′	
qPCR	*gdh*F1	5′-GGGCAAGTCCGACAACGA -3′	^51^
	*gdh*R1	5′-GCACATCTCCTCCAGGAAGTAGAC-3′	
	Probe	5′-TCATGCGCTTCTGCCAG BHQ2–3′	
***Giardia tpi***	Primary reaction		^54^
	AL3543	5′-AAATIATGCCTGCTCGTCG-3′	
	AL3546	5′-CAAACCTTITCCGCAAACC-3′	
	Secondary reaction		
	AL3544	5′-CCCTTCATCGGIGGTAACTT-3′	
	AL3545	5′-GTGGCCACCACICCCGTGCC-3′	

The presence of *Giardia* in all samples were screened at the glutamate dehydrogenase (*gdh*) locus using a quantitative PCR (qPCR) procedure previously described by Yang *et al*.^51^. Conventional PCR amplification of the glutamate dehydrogenase (*gdh*) and the triose phosphate isomerase (*tpi*) locus was conducted on all samples found positive for *Giardia* on qPCR. An approximately 733 bp portion of the *gdh* gene was obtained using formerly published primers^52,53^. For this nested PCR, primers *GDH*eF and *GDH* 2 were used in the primary reaction, and primers *GDH*iF and *GDH* 4 were used in the secondary reaction (Table 2). PCR reaction volume for each sample in both primary and secondary PCRs was 20 µL, containing 10 µL GoTaq® Green Master Mix (Promega,USA), 0.25 µM of each primer, 4 µL nuclease-free water and 5 µL of template genomic DNA. Cycling conditions for primary PCR were 1 cycle of 94 °C for 2 min, followed by 35 cycles of 94 °C for 30 s, 55 °C for 30 s and 72 °C for 60 s with a final extension of 72 °C for 7 min and a 12 °C hold. The cycling conditions for the secondary PCR were similar to the primary PCR, except the annealing temperature, which was 52 °C. PCR of the *tpi* locus utilised a nested PCR protocol developed by Sulaiman *et al*. (2003) with slight modifications^54^. Primary and secondary primers are shown in Table 6. The predicted PCR product sizes of primary and secondary reactions were 605 bp and 530 bp, respectively. The PCR reaction for the primary reaction comprised of: 10 µL GoTaq® Green Master Mix (Promega,USA), 0.25 µM of each primer, 4 µL nuclease-free water and 5 µL of template DNA. The secondary reaction contained: 12.5 µL GoTaq® Green Master Mix (Promega,USA), 0.25 µM of each primer, 6.25 µL nuclease-free water and 5 µL of DNA. The following cycling conditions were used for both primary and secondary PCRs: 1 cycle of 94 °C for 2 min, followed by 35 cycles of 94 °C for 45 s, 60 °C for 45 s and 72 °C for 1 min with a final extension of 72 °C for 7 min. The amplified DNA products from the *gdh* and *tpi* PCR were visualized on a 1.5% agarose gel containing SYBR®Safe DNA gel stain.

PCR products of the *Ancylostoma spp*., *Giardia gdh* and *Giardia tpi* reactions were excised from gels and purified using the Wizard®SV Gel and PCR Clean-Up System kit (Promega, USA) before DNA sequencing. DNA sequencing was performed at the Australian Genome Research Facility (Perth, WA).

Following screening by qPCR for *Giardia*, only samples which were positive upon Sanger sequencing on the *gdh* and/or *tpi* assays were considered as confirmed positives. *Ancylostoma* positive status was also based on Sanger sequence positive PCR results.

### Data availability

The datasets generated during and/or analysed during the current study are available from the corresponding author on reasonable request.

### Accession codes

B1 B12 *gdh* (accession number: MF 990015), B1 B12 *tpi* (accession number: MF 974557), B2 B18 *gdh* (accession number: MF 990016), B2 B25 *tpi* (accession number: MF 974558), B1 B5 *gdh* (accession number: MF 769400),B1 B5 *tpi* (accession number: MF 974555), B1 B7 *gdh* (accession number: MF 990014), B1 B7 *tpi* (accession number: MF 974556), B1 HC10 *gdh* (accession number: MF 990017), B1 HC23 *tpi* (accession number: MF 974560), B1 HC34 *gdh* (accession number: MF 990018), B1 HC7 *tpi* (accession number: MF 974559).
